# Modification of Cellulose with Succinic Anhydride in TBAA/DMSO Mixed Solvent under Catalyst-Free Conditions

**DOI:** 10.3390/ma10050526

**Published:** 2017-05-12

**Authors:** Ping-Ping Xin, Yao-Bing Huang, Chung-Yun Hse, Huai N. Cheng, Chaobo Huang, Hui Pan

**Affiliations:** 1 College of Chemical Engineering, Nanjing Forestry University, 159 Longpan Road, Nanjing 210037, China; smallxin1992@163.com (P.-P.X.); hyb123@mail.ustc.edu.cn (Y.-B.H.); Chaobo.HUANG@njfu.edu.cn (C.H.); 2 Southern Research Station, USDA Forest Service, Pineville, LA 71360, USA; chse@fs.fed.us; 3 USDA Agricultural Research Service, Southern Regional Research Center, 1100 Robert E. Lee Blvd., New Orleans, LA 70124, USA; hn.cheng@ars.usda.gov

**Keywords:** succinoylation, ionic liquid, cellulose, modification, adsorption

## Abstract

Homogeneous modification of cellulose with succinic anhydride was performed using tetrabutylammonium acetate (TBAA)/dimethyl sulfoxide (DMSO) mixed solvent. The molar ratio of succinic anhydride (SA) to free hydroxyl groups in the anhydroglucose units (AGU), TBAA dosage, reaction temperature, and reaction time were investigated. The highest degree of substitution (DS) value of 1.191 was obtained in a 10 wt% TBAA/DMSO mixed solvent at 60 °C for 60 min, and the molar ratio of SA/AGU was 6/1. The molar ratio of SA/AGU and the TBAA dosage showed a significant influence on the reaction. The succinoylated cellulose was characterized by ATR-FTIR, TGA, XRD, solid state CP/MAS ^13^C NMR spectroscopy (CP/MAS ^13^C NMR), and SEM. Moreover, the modified cellulose was applied for the adsorption of Cu^2+^ and Cd^2+^, and both the DS values of modified cellulose and pH of the heavy metal ion solutions affected the adsorption capacity of succinylated cellulose. The highest capacity for Cu^2+^ and Cd^2+^ adsorption was 42.05 mg/g and 49.0 mg/g, respectively.

## 1. Introduction

Cellulose is the most abundant natural polymer on earth. It has several unique properties such as biocompatibility, biodegrability, low cost, and chemical stability, which makes it a promising resource to replace fossil resources for the production of industrial materials, chemicals, and biofuels [1,2,3,4]. Cellulose-based materials have attracted much attention during the past few decades and are widely used in adhesives, food, spinning, and biomedical materials [5,6,7]. The chemical modification of the hydroxyl groups on cellulose is one important approach to tailor the structure and properties of cellulose by introducing new functional groups so that the obtained cellulose derivatives can meet the requirements of specific applications [5]. Conventional modification approaches include sulfonation [8], esterification [9,10], etherification [11,12], and silylation [13].

While many studies have reported success in the chemical modifications of cellulose, limitations or drawbacks can still be found when considering some of these systems. Cellulose is insoluble in water and most organic solvents under conventional conditions due to its rich-in-oxygen nature and highly crystalline regions [14,15,16]. Therefore, an inadequate dispersion in reaction media, low degree of substitution (DS) values, long reaction times, and side reactions were often observed during cellulose modification in heterogeneous systems [17]. To address these issues, new cellulose solvents were used to dissolve cellulose and provided more free –OH groups to react with the modification reagents. Alkylimidazolium-based ILs (i.e., AmimCl, EminAc, and BminCl) have been the most frequently used cellulose solvents [18]. Other multi-component systems for cellulose dissolution, such as DMAc/LiCl [19,20] and DMSO/TBAF [21,22], have also been reported. The formation of a homogeneous cellulose solution facilitates the easy and homogeneous modification of cellulose with different functional groups and offers different cellulose-based materials such as cellulose acetate, propionate, butyrate, pentanoate, hexanoate, and laurate [23,24,25,26].

The succinoylation of cellulose has been widely used to introduce ester groups on cellulose and to form linkages with terminal free fatty acid groups. Succinylated cellulose has a wide range of applications [27,28,29], such as adsorbents for water and oil, medicines for drug delivery systems, and as thermoplastic materials [30,31,32]. Generally, the succinoylation is catalysed by basic catalysts such as triethylamine and 4-Dimethylaminopyridine (DMAP), which facilitates the deprotonation of –OH groups and accelerates the reaction. For example, Yin et al. used *N*,*N*-dimethylaetamide/LiCl solvent to dissolve bacterial cellulose and then modified it with succinic anhydride over a triethylamine catalyst. A high DS value of 1.45 was achieved under the optimized reaction conditions [33]. Sun et al. selected DMAP, an effective catalyst for the acylation of alcohol, combined with [BMIM]Cl, to prepare succinylated cellulose and achieved the highest DS value of 2.34 under 100 °C [34]. Similarly, Chen et al. used the same catalyst for the homogeneous modification of cellulose in tetraethylammonium chloride (TEACl)/dimethyl sulfoxide (DMSO) and tetra butylammonium fluoride (TBAF)/dimethyl sulfoxide (DMSO) mixed solvents, with the DS values ranging from 0.41 to 2.11 [35,36]. Moreover, other catalysts, such as iodine and *N*-bromosuccinimide (NBS), were also found to exhibit good catalytic activity in the succinoylation of cellulose [37].

In recent years, the non-catalytic system has also raised considerable attention, which can greatly simplify the reaction process, and some catalyst-free systems have been reported. For example, Geng et al. used a 1-butyl-3-methylimidazolium chloride ionic liquid ([C4 min]Cl)/DMSO system for cellulose succinoylation and the DS value ranged from 0.037 to 0.53 [38]. Liu et al. used 1-allyl-3-methylimidazolium Chloride ([Amim]Cl) as a solvent for a succinoylation reaction and achieved low DS values ranging from 0.071 to 0.22 [39]. Similar results were obtained by using 1-buty-3-methylimidazolium ([BMIM]Cl) as a reaction solvent for the succinoylation reaction [40]. The aromatic heterocyclic cation of these ionic liquids acted as the catalysts, which could thereby simplify the reaction system. However, it is not difficult to observe that the DS values of these systems were still very low.

Recently, we reported a TBAA/DMSO mixed solvent for the dissolution of cellulose at ambient temperature [41]. The cations and anions were found to easily interact with the –OH groups on the cellulose chain and thereby disrupted inter- and intramolecular hydrogen bonding. This solvent possesses great potential to be used for the homogeneous chemical modification of cellulose. Moreover, to the best of our knowledge, no TBAA/DMSO solvent has been reported for the succinoylation of cellulose. Moreover, the nitrogen-containing NR_4_^+^, as well as the basic nature of TBAA, may also serve as a catalyst for the succinoylation conversion. By realizing these two aspects, we studied the succinoylation of cellulose in a TBAA/DMSO mixed solvent, without an additional catalyst.

**Scheme 1:**
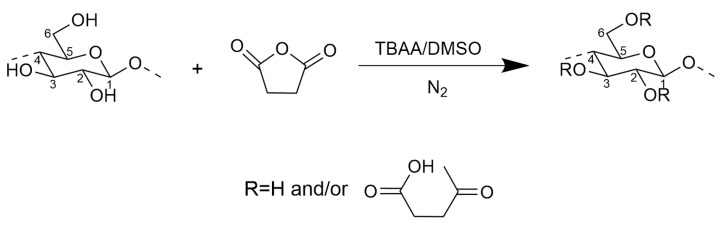
Succinoylation of cellulose with succinic anhydride in TBAA/DMSO mixed solvent.

Herein, we report on the succinoylation of cellulose in a TBAA/DMSO mixed solvent. A high DS value of 1.191 was obtained without any catalyst (Scheme 1). This DS value was much higher than that from the reported literature. In this study, the effects of the mole ratio of succinic anhydride (SA) to free hydroxyl groups in the anhydroglucose units (AGU), TBAA dosage, reaction temperature, and reaction time on the homogeneous succinoylation of cellulose were investigated. The succinylated cellulose was characterized by ATR-FTIR, TGA, Solid state CP/MAS ^13^C NMR, XRD, and SEM. The adsorption capacities for Cu^2+^ and Cd^2+^ of succinylated cellulose were also studied.

## 2. Results and Discussion

### 2.1. Succinylation of Cellulose

**Figure 1:**
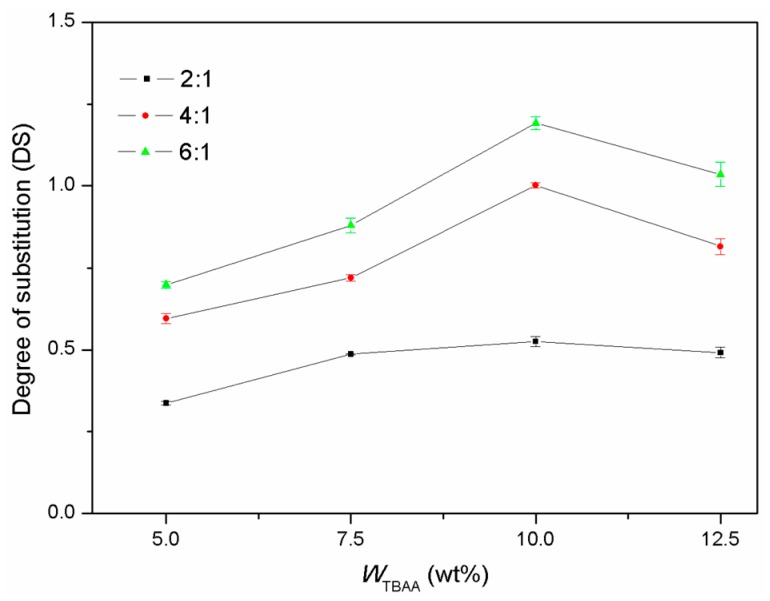
DS values of succinylated cellulose prepared using different TBAA/DMSO mixed solvents and with different weights of succinic anhydride. The molar ratio of succinic anhydride/anhydroglucose in cellulose was 2:1, 4:1, 6:1.

Firstly, the TBAA/DMSO mixed solvent was used to dissolve cellulose and the succinylation process was then carried out by adding SA to the mixed system. Figure 1 shows the DS values of succinylated cellulose modified at different TBAA dosage and succinic acid to AGU ratios. In general, the DS values of succinylated cellulose initially increased when *W*_TBAA_ was increased from 5.0 wt% to10.0 wt%, and then decreased as *W*_TBAA_ was further increased to 12.5 wt%. The effect of the TBAA dosage on the homogeneous succinylation of cellulose was related to the degree of cellulose dissolution in the mixed solvent [35]. Our previous study showed that both the solubility and dissolution rate of cellulose in the TBAA/DMSO mixed solvent increased as the TBAA dosage increased in the mixed solvent, until it reached 15 wt% [41]. In this regard, the accessibility of succinic anhydride to the hydroxyl groups on dissolved cellulose would be improved as more cellulose dissolved and more hydroxyl groups were freed, resulting in an increase in the DS value of the modified cellulose. However, further increasing the dosage of TBAA also increased the viscosity of the mixed solvent, which negatively affected the mass transfer of the reaction system and inhibited the succinoylation reaction [42,43]. In addition, a high TBAA dosage in the mixed solvent could also bring a steric hindrance due to the relatively large cation structure of TBAA, which may prevent SA from attacking the hydroxyl groups on cellulose. Similar results have been reported by other research [35,36].

The mole ratio of SA/AGU also had a significant effect on the succinoylation of cellulose. The DS values of modified cellulose increased as the concentration of SA increased, especially when the SA/AGU ratio increased from 2/1 to 4/1. Further increasing the SA/AGU ratio to 6/1 only provided a small increment in the DS values. When considering the atom economy, a lower SA/AGU ratio was preferable. Therefore, a mole ratio of SA/AGU of 4/1 and *W*_TBAA_ = 10.0 wt% were selected for further study. Other reaction parameters such as reaction temperature and reaction time were also optimized, and the results are listed in Table S1. In general, the results in Table S1 show that the DS values of succinylated cellulose prepared in the TBAA/DMSO mixed solvent ranged from 0.337 to 1.191, which were much higher than those of succinylated cellulose prepared in other catalyst-free systems (i.e., 0.037~0.53, 0.071~0.22) [38,39]. Therefore, the TBAA/DMSO mixed solvent had a much higher reactivity toward the modification of cellulose with succinic anhydride.

### 2.2. ATR-FTIR Analysis

**Figure 2:**
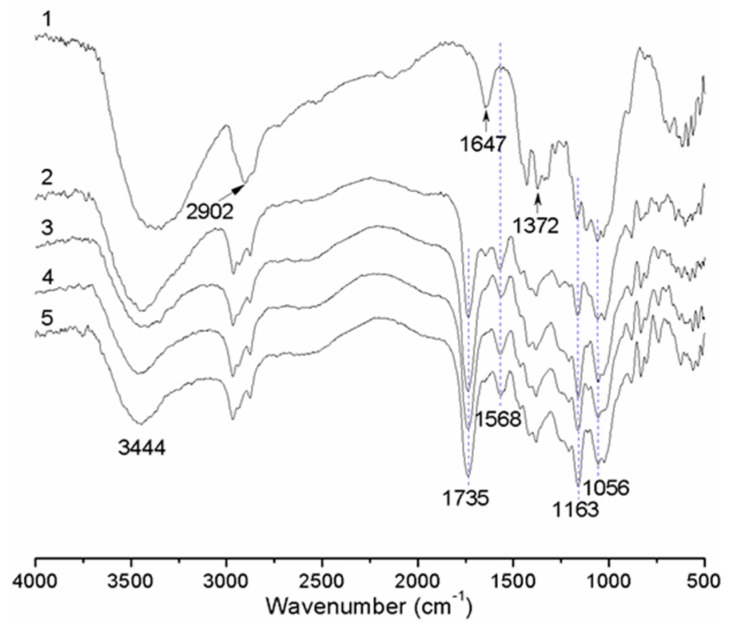
ATR-FTIR spectra of unmodified cellulose (spectrum 1) and succinylated cellulose prepared with the *W*_TBAA_ at 5.0 wt% (spectrum 2), 7.5 wt% (spectrum 3), 10.0 wt% (spectrum 4), and 12.5 wt% (spectrum 5).

Figure 2 shows the ATR-FTIR spectra of unmodified cellulose (spectrum 1) and succinylated cellulose prepared with different *W*_TBAA_ (spectrum 2–5). The peaks at 3444, 2902, 1647, 1372, and 1056 cm^−1^ are associated with native cellulose, where: the broad peak at 3444 cm^−1^ corresponds to the O–H stretching; the peak at 2902 cm^−1^ is generated by the C–H stretching of CH_2_; the absorption at 1647 cm^−1^ is attributed to the moisture in the cellulose; and the peaks at 1372 and 1056 cm^−1^ are from O–H bending and C–O–C pyranose ring skeletal vibration, respectively [36,44]. Compared to the unmodified cellulose, two new peaks at 1735 and 1568 cm^−1^ were found in all modified cellulose samples. The band at 1735 cm^−1^ is assigned to the carbonyl groups in –COOH or –COOR, while the band at 1568 cm^−1^ is characterized as the antisymmetric stretching of carboxylic anions, providing the evidence of successful succinoylation [40]. Furthermore, the intensity of the absorption band at 1163 cm^−1^, attributed to C–O antisymmetric bridge stretching in esters, increased after succinoylation. It is likely caused by the formation of an ester or carboxylic acid [34]. The appearance of all the new peaks confirmed the successful succinoylation of cellulose in the TBAA/DMSO mixed solvent. In addition, the absence of peaks at 1850 and 1780 cm^−1^ in the spectra of succinylated cellulose samples indicated that un-reacted succinic anhydride had been removed from the modified cellulose [34].

It was also worth noting that the intensity of the peaks of the ester group (i.e., 1735, 1568, and 1163 cm^−1^) initially increased as *W*_TBAA_ increased from 5.0 wt% to 10.0 wt%, and then decreased slightly when the TBAA dosage was further increased to 12.5 wt%. This result was in good agreement with the change of the DS values of succinylated cellulose, which increased from 0.595 to 1.002 when *W*_TBAA_ increased from 5.0 to10.0 wt% and then decreased to 0.815 as *W*_TBAA_ increased to 12.5 wt%. Corresponding to the DS values shown in Table S1, the intensity of the peaks at 1735, 1568, and 1163 cm^−^^1^ displayed no visible differences when the reaction temperature ranged from 20 °C to 80 °C (Figure S1) or when the reaction time ranged from 30 min to 90 min (Figure S2).

### 2.3. TGA Analysis

**Figure 3:**
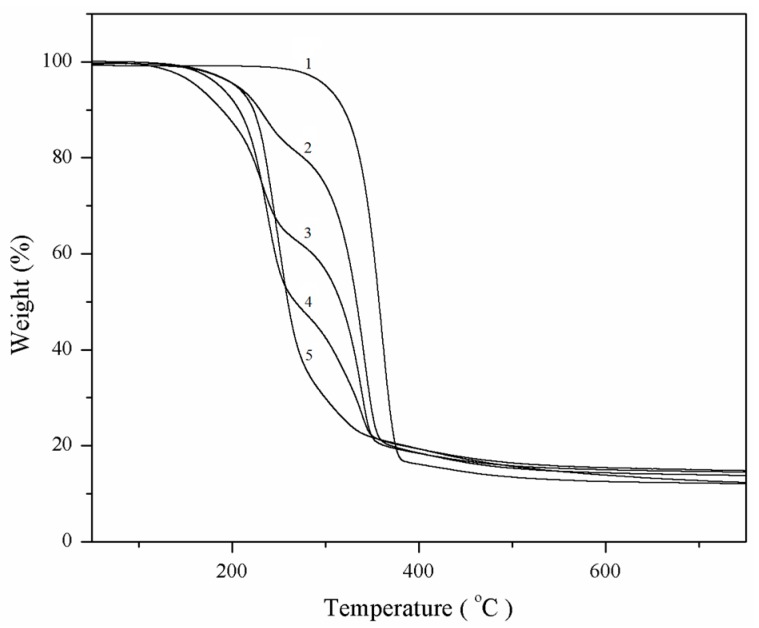
The thermograms spectra of unmodified (spectrum 1) and succinylated cellulose prepared with the *W*_TBAA_ at 5.0 wt% (spectrum 2), 7.5 wt% (spectrum 3), 10.0 wt% (spectrum 4), and 12.5 wt% (spectrum 5).

The thermal properties of unmodified cellulose and succinylated cellulose were investigated and the results are shown in Figure 3. It can be seen that the onset decomposition temperature of unmodified cellulose was around 240 °C, and the succinylated cellulose samples started to decompose at about 141 °C, depending on the particular cellulose sample modified in a different dosage of TBAA. This result was due to the formation of cellulose esters, which was believed to lower the thermal stability of cellulose [45].

The TGA curves of the succinylated cellulose samples all displayed an obvious two-stage decomposition pattern, especially for those samples modified at *W*_TBAA_ from 5.0 to 10.0 wt%. This result implied that succinylated cellulose was a mixture of two types of cellulose, i.e., native and regenerated cellulose. The first stage should be ascribed to the decomposition of regenerated cellulose, which has been previously reported [46]. The second stage belonged to native cellulose. According to our previous work, the solubility of cellulose increased as the TBAA dosage increased in the mixed solvent [41], which left less native cellulose in the reaction mixture after succinoylation. Thus, the decomposition temperature gradually reduced as the TBAA dosage increased from 5.0 wt% to 12.5 wt%. These results further confirmed the important role of TBAA dosage on the DS value of the succoylation reaction.

### 2.4. CP/MAS ^13^C NMR Analysis

**Figure 4:**
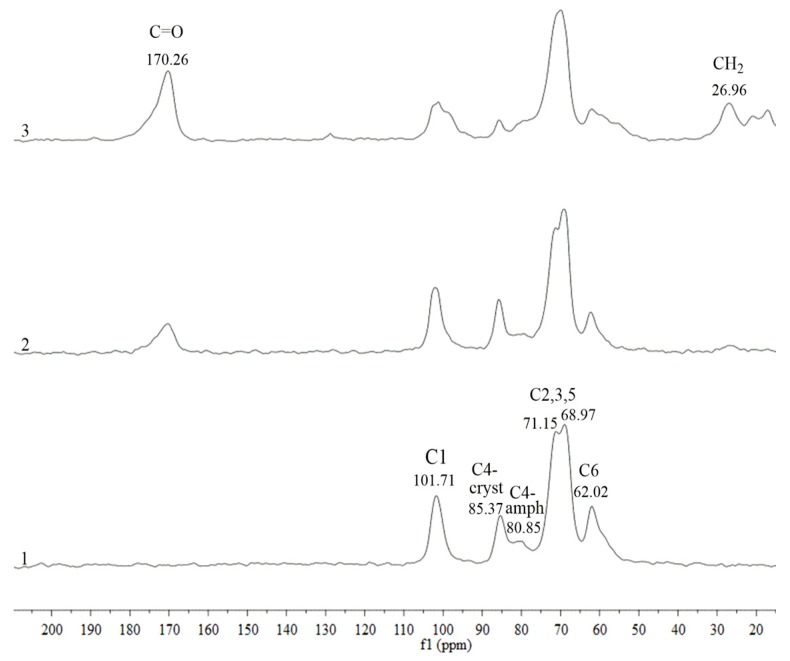
Solid-state CP/MAS ^13^C NMR spectra of unmodified (spectrum 1) cellulose and succinylated cellulose prepared with the *W*_TBAA_ at 5.0 wt% (spectrum 2), and 10.0 wt% (spectrum 3).

The solid-state CP/MAS ^13^C NMR spectra of unmodified cellulose and succinylated cellulose prepared with two typical TBAA dosages are shown in Figure 4. The chemical shift between 50 to 110 ppm was assigned to the carbons on cellulose. Specifically, the signals at around 85.37 and 80.85 were assigned to the C-4 on crystalline and amorphous cellulose, respectively [45,47]. Compared to unmodified cellulose, the spectra of succinylated cellulose displayed two new signals. The peak at 170.26 ppm was attributed to the carbonyl carbon, while the signal at 26.96 ppm was attributed to the methylene group on a cellulose succinoyl ester. This result suggested that part of the –OH was converted to a succinoyl ester group, indicating the successful succinoylation of cellulose. In addition, the intensities of these two signals slightly increased as the TBAA dosage increased from 5.0 to 10.0 wt% (spectra 2 and 3). This result was consistent with the DS values of succinylated cellulose samples (Figure 1), which suggested an increase in the reactivity of cellulose when the TBAA dosage increased. Similar results were also reported by other studies [45].

After succinoylation, the signals at 80.85 and 85.37 ppm (C-4 on cellulose) became weak (spectrum 2 and 3), which indicated that both the amorphous and crystalline region of cellulose reacted during the succinoylation process [47,48]. It is known that the hydroxyls of C-2, C-3, and C-6 are the three main reactive sites on cellulose. After the reaction, the signal of C-6 displayed a significant decrease of succinylated cellulose as compared to the signals of C-2 and C-3 (spectra 3). This result implied that SA prefers to react with the hydroxyl on C-6 rather than those on C-2 and C-3 [23,45].

### 2.5. XRD Analysis

**Figure 5:**
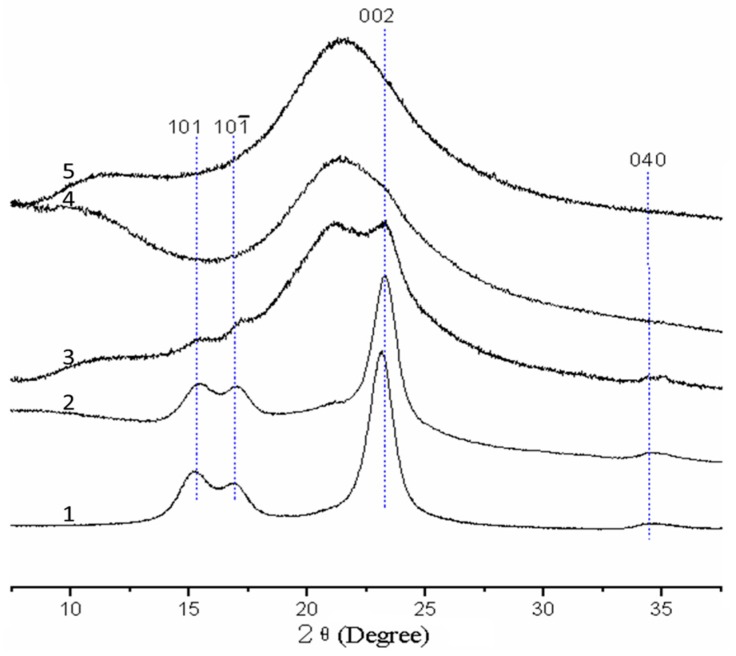
X-ray diffraction spectra of unmodified (spectrum 1) cellulose and succinylated cellulose prepared with the *W*_TBAA_ at 5.0 wt% (spectrum 2), 7.5 wt% (spectrum 3), 10.0 wt% (spectrum 4), and 12.5 wt% (spectrum 5).

The X-ray diffraction curves of unmodified cellulose and succinylated cellulose are shown in Figure 5. The pattern of unmodified cellulose and the succinylated cellulose prepared with *W*_TBAA_ at 5.0 wt% were similar (spectra 1 and 2). They both displayed a typical diffraction pattern of cellulose I. The diffraction peaks at 14.8°, 16.4°, 22.7°, and 34.2° were attributed to 101, 10$\bar{1}$, 002, and 040 planes, respectively [49,50]. No cellulose dissolution is expected for *W*_TBAA_ of 5.0 wt% and below. On the other hand, succinylated cellulose prepared at a high TBAA ratio had a board peak at around 20.9° (spectra 4 and 5), which was a typical diffraction pattern of cellulose II. It indicated that most of cellulose underwent the succinoylation process when it was dissolved in the TBAA/DMSO mixed solvent. A small amount of native cellulose I still remained in the succinylated cellulose sample prepared with *W*_TBAA_ at 10.0 wt%, as indicated in the TGA analysis. However, it was not found in the XRD result, which is likely due to the weak signal of the small amount of cellulose I in this sample. The diffraction pattern of the succinylated cellulose sample prepared with *W*_TBAA_ at 7.5 wt% clearly showed the coexistence of native cellulose I and regenerated cellulose II, which displayed both diffraction planes of cellulose I and II (spectrum 3).

### 2.6. SEM Analysis

**Figure 6:**
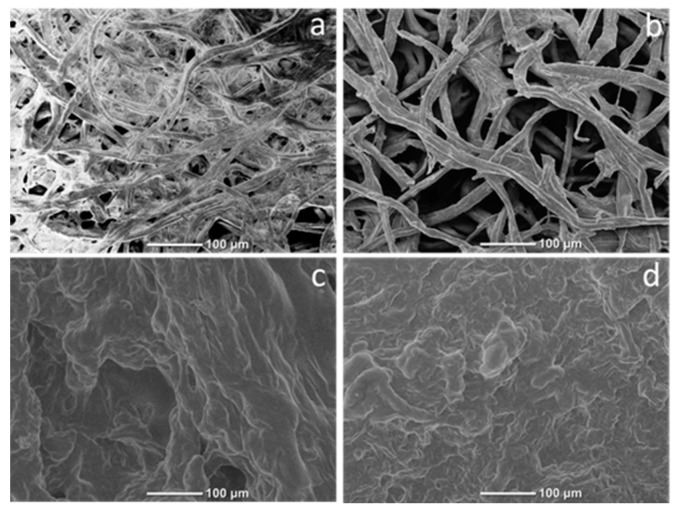
SEM images of unmodified cellulose and succinylated cellulose: (**a**) original filter paper and modified cellulose in TBAA/DMSO mixed solvents with the TBAA dosage of (**b**) 5.0 wt%; (**c**) 10.0 wt%; (**d**) 12.5 wt%.

The morphology of unmodified cellulose and succinylated cellulose are presented in Figure 6. Additionally, the pictures of unmodified cellulose and succinylated cellulose are also shown in Figure S3. It can be seen that both unmodified cellulose (Figure 6a) and succinylated cellulose prepared from the TBAA/DMSO mixed solvent with a TBAA dosage at 5.0 wt% (Figure 6b) remained fiberous in appearance, except that the succinylated cellulose fiber exhibited a smoother surface. This result inferred that the dissolution and/or modification of the cellulose fiber started from the surface of the cellulose fiber [28]. For those fibrils on the surface of fibers (i.e., which caused the “fuzzy” and rough appearance on the surface of unmodified cellulose fiber), they were more accessible to the solvent and reagent during the dissolution and succinoylation processes and would react earlier than the inner part of the fiber. Therefore, the result provided a smooth surface of succinylated cellulose. Succinylated cellulose prepared at higher TBAA dosages displayed a homogeneous appearance of regenerated cellulose (Figure 6c,d). Although the succinylated cellulose sample prepared in 10.0 wt% TBAA still contained a small amount of native cellulose (as indicated in TGA analysis), no fiberous cellulose was observed in this sample. This is probably due to the coverage by the large amount of regenerated cellulose.

### 2.7. Application for the Adsorption of Heavy Metal Ions

The succinylated cellulose was used for the adsorption of heavy metal ions in aqueous solutions. The tests were conducted using succinylated cellulose of different DS values in an aqueous metal ion solution at 25 °C for 10 min. Both Cu^2+^ and Cd^2+^ ions will easily form their hydroxide precipitate when the pH values are above 5.0. Therefore, the pH of all the heavy metal ion solutions studied in this work were below 5.0.

**Figure 7:**
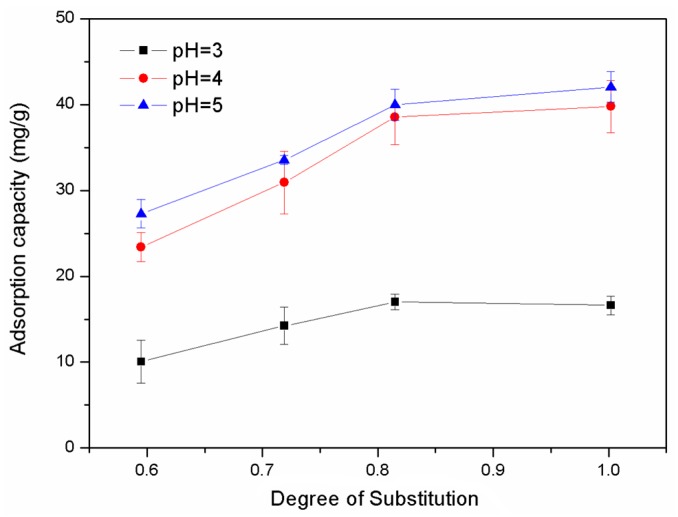
Effect of DS and pH on the adsorption capacity of Cu^2+^.

As shown in Figure 7, the DS values had a great influence on the metal adsorption capacity of succinylated cellulose. When the pH of the Cu^2+^ solutions was 4.0 or 5.0, the adsorption capacity of succinylated cellulose increased as the DS values increased from 0.595 to 1.002. This indicated that the metal ions were gradually absorbed as more carboxyl groups (–COOH) were grafted on the surface of the cellulose. The adsorption capacities increased almost linearly with the DS values when the DS values were below 0.8, indicating that the adsorption sites on the succinylated cellulose were sufficient for metal ion adsorption. However, a further increase of the DS values only provided a slight increase in the adsorption capacity, which may be caused by the equilibrium of the metal ions in solution and on the cellulose surface. [33,51]. The highest capacity for Cu^2+^ adsorption (42.05 mg/g) was achieved by using the modified cellulose with a DS value of 1.002 when the pH value of the solution was 5.0. However, when the solution’s pH was 3.0, the adsorption capacity of succinylated cellulose initially increased as the DS values ranged from 0.595 to 0.815 and then slightly decreased as the DS value was further increased to 1.002.

It is worth noting that all of the succinylated cellulose provided a higher capacity to absorb metal ions as the pH value of the solution increased. This is because the carboxyl groups retained the protons in the solution at a lower pH, and its capacity to bind the positively charged ions decreased. However, at a high pH (4.0 or 5.0), the carboxyl groups were apt to deprotonate in the solution and existed as –COO^−^, which can more effectively bond to the positively charged metal ions. Both the protons and the metal ions in the solutions can bond with the carboxyl groups, mainly depending on the pH values of the solutions [52,53].

**Figure 8:**
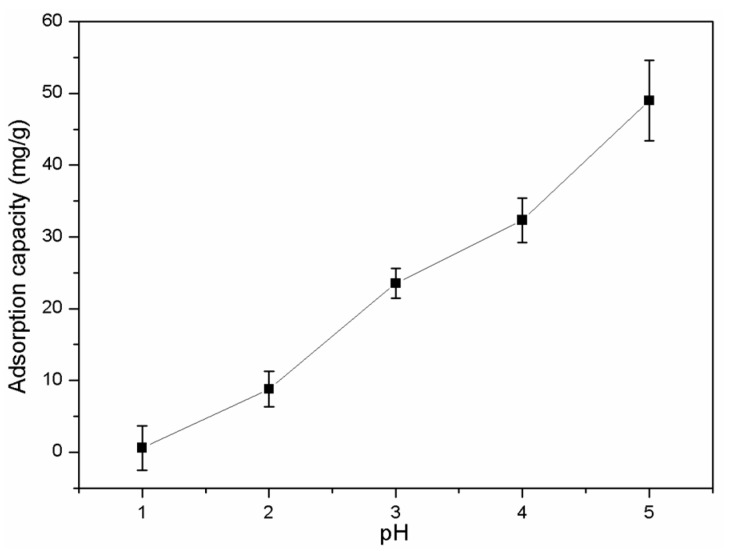
Effect of pH on the adsorption of Cd^2+^ with succinylated cellulose (DS = 1.002).

The adsorption of Cd^2+^ with succinylated cellulose (DS = 1.002) was also investigated as the pH value ranged from 1.0 to 5.0 (Figure 8). The results presented a similar adsorption tendency to that of Cu^2+^, and the adsorption of Cd^2+^ increased from 0.6 mg/g to 49.0 mg/g when the pH values of the solutions increased from 1.0 to 5.0. According to the above results, both the Cu^2+^ and Cd^2+^ ions can be efficiently absorbed by the succinylated cellulose, demonstrating the high potential of succinylated cellulose as an absorbent for the removal of heavy metal ions in the environment.

## 3. Materials and Methods

### 3.1. Materials

Cellulose filter papers (diameter 125 mm, Whatman, Hangzhou, China) were cut into (2 × 5 mm) pieces and dried overnight at 105 °C before use. Tetrabutylammonium acetate (Sigma-Aldrich, Santa Clara, CA, USA, 97%), Dimethyl sulfoxide (BDH, 99.7%), Succinic anhydride (Alfa Aesar, Shanghai, China, 99%), and Isopropyl Alcohol (BDH, Radmanovic, PA, USA, 99.5%) were used without further purification. All other chemicals were purchased from commercial sources and were of reagent grade.

### 3.2. Succinoylation of Cellulose with Succinic Anhydride in TBAA/DMSO Mixed Solvent

The succinoylation of cellulose was carried out by the homogeneous reaction of cellulose filter paper in TBAA/DMSO mixed solvent. Four mixed solvents were prepared with TBAA dosages in the mixed solvent at 5.0 wt%, 7.5 wt%, 10.0 wt%, and 12.5 wt% and were denoted as *W*_TBAA_ = 5.0 wt%, 7.5 wt%, 10.0 wt%, and 12.5 wt%, respectively. A typical succinoylation procedure of cellulose was as follows: Approximately 1.60 g of dried cellulose filter paper was added to 8.00 g of homogeneously mixed solvent in a 100 mL round bottom flask. The cellulose/TBAA/DMSO mixtures were stirred at 60 °C for 30 min. The filter paper was well dissolved and achieved a homogeneous mixture. Then, solid succinic anhydride was added into the mixture and swept with N_2_ to ensure that the air in the round bottom was removed. Afterword, the mixture was sealed with a rubber stopper and then the homogeneous modification was carried out at different reaction temperatures with magnetic stirring. After a certain of reaction time, 50 mL isopropyl alcohol was slowly added into the resulting mixture with continuous stirring at room temperature for 2 h. The solid precipitant was filtered and thoroughly washed with isopropyl alcohol to guarantee that TBAA, DMSO, un-reacted succinic anhydride, and any by-products were eliminated. Finally, the solid was dried under vacuum at 50 °C overnight.

### 3.3. Characterization

#### 3.3.1. Determination of the Degree of Substitution (DS)

In the present study, succinylated cellulose was prepared using TBAA/DMSO mixed solvents without a catalyst, and the reaction route is shown in Scheme 1. The degree of substitution (DS) of succinylated cellulose was determined by a direct titration method [54,55]. In brief, a known weight of succinylated cellulose (0.06 g) was placed in a flask, followed by the addition of 10 mL of NaOH (0.1 mol·L^−1^). The mixture was allowed to react at 50 °C for 30 min with magnetic stirring. At the end of the reaction time, any excess amount of NaOH was titrated by a standard HCl solution (0.025 mol·L^−1^). All the experimental data were repeated three times. The DS value was calculated by the following equation:

(1) $$\mathrm{DS}\;=\frac{162\times n_{\mathrm{COOH}}}{m-100\times n_{\mathrm{COOH}}}$$

where 162 (g/mol) is the molar mass of an anhydroglucose unit (AGU); 100 (g/mol) is the net increase in the mass of an AGU for each succinoylation substituted; and m is the weight of the sample for every experiment. *n*_COOH_ is the amount of COOH and calculated by Equation (2), where V_NaOH_ is the volume of standard NaOH solution in the titration; C_NaOH_ is the concentration of standard NaOH; V_HCl_ is the volume of standard HCl consumed in the titration; and C_HCl_ is the molarity of standard HCl solution.

(2) $$n_{\mathrm{COOH}}=\left(V_{\mathrm{NaOH}}\times C_{\mathrm{NaOH}}-V_{\mathrm{HCl}}\times C_{\mathrm{HCl}}\right)/2$$

#### 3.3.2. Attenuated Total Reflectance-Fourier Transform Infrared Spectroscopy (ATR-FTIR) Analysis

ATR-FTIR characterization of cellulose samples was performed on a Thermo Nicolet 360 (Nicolet, Madison, WI, USA) coupled with a Spectra Golden Gate MKII Single Reflection ATR accessory. The FTIR analyses were conducted between a frequency range of 4000–400 cm^−1^.

#### 3.3.3. Thermal Gravimetric (TGA) Analysis

The thermal stability of cellulose samples was characterized using a TA Instrument Q 500 (TA Instruments, New Castle, DE, USA). Cellulose samples weighing between 2 and 5 mg were tested in each run. The cellulose sample was heated from room temperature to 800 °C, at a heating rate of 10 °C/min under nitrogen flow (40 mL/min).

#### 3.3.4. Solid State CP/MAS ^13^C NMR Spectroscopy (CP/MAS ^13^C NMR) Analysis

The solid-state CP/MAS ^13^C NMR spectra were recorded on a Bruker AVIII-400 MHz with a 4 mm HR MAS BL_4_ probe. The spectroscopy of cellulose samples for solid state CP-MAS (cross-polarisation, magic anglespinning) ^13^C NMR spectroscopy was obtained on a Bruker AVIII spectrometer (Bruker, Germany) and the span rate of the 4 mm rotor was 12 kHZ for the test. The acquisition time was 0.0127 s, and the recycle delay was 2.0 s.

#### 3.3.5. X-ray Diffraction (XRD) Analysis

The X-ray diffraction measurements of cellulose samples were analyzed using wide angle X-ray diffraction (Rigaku Ultima IV, Kyoto, Japan). Patterns were acquired with Cu Kα radiation (λ = 1.5406 Å) at 40 kV and 30 mA over the 2θ range from 5 to 40° at a scan speed of 2 deg·min^−1^.

#### 3.3.6. Scanning Electron Microscopy (SEM) Analysis

The surface morphology of the cellulose samples was observed using scanning electron microscopy (SEM JCM-5000, Kyoto, Japan). Each sample was coated with gold using a vacuum sputter coater for 60 s, before being subjected to the SEM analysis.

### 3.4. Metal Ion Adsorption Test

The metal-adsorption capacity of the succinylated cellulose (sample 2 DS = 0.595, sample 5 DS = 0.719, sample 8 DS = 1.002, sample 11 DS = 0.815) was determined according to the following method. A total of 0.02 g succinylated cellulose was placed in a 100 mL beaker and then mixed with 10 mL metal ion solution (Cu^2+^ 128 mg/L, Cd^2+^ 300 mg/L) by stirring at 25 °C for 10 min. The pH of the Cu^2+^ ions solution ranged from 3.0 to 5.0, and 1.0 to 5.0 for Cd^2+^. The pH was adjusted with HCl or NaOH solutions at 0.01~0.1 mol/L. After adsorption for 10 min, the solution was filtered, and the metal ion concentration was determined by atomic adsorption spectroscopy (Perkin Elmer, AA900T Waltham, MA, USA). All of the experiments were repeated three times.

## 4. Conclusions

This study reported a TBAA/DMSO mixed solvent for the succinoylation of cellulose under a catalyst-free condition. The highest DS value of 1.191 was obtained in a 10.0 wt% TBAA/DMSO solvent at 6/1 SA/AGU, 60 °C, for 60 min. The DS values of succinylated cellulose ranged from 0.337 to 1.191 while differing the reaction conditions. The succinoylation reaction was also significantly affected by the TBAA dosage, which played the role of the solvent and catalyst in the whole reaction process. ATR-FTIR and solid-state CP/MAS ^13^C NMR spectroscopies confirmed the successful succinoylation of cellulose in the TBAA/DMSO mixed solvent. The thermal stability of cellulose decreased after the succinoylation. XRD analysis of the crystal of succinylated cellulose indicated that it changed from cellulose I to cellulose II. Moreover, these modified cellulose products showed good adsorption capacities for Cu^2+^ and Cd^2+^ ions in aqueous solutions. The successful modification of cellulose with SA not only provides a new mild method for the homogeneous succinylation of cellulose under a catalyst-free condition, but also demonstrates the unique role of quaternary ammonium type ionic liquid in catalyzing the esterification of the hydroxyl groups, which might be a guide for the designing of more rational catalyst-free systems for the modification of cellulose with other chemical reagents. Besides, the obtained succinylated cellulose materials showed a good absorption capacity of the metal cations, which may find important applications in the water pollution treatment in the future. Future work will be focused on the study of more cellulose products based on the homogeneous modification of cellulose in the TBAA/DMSO system and the recycling of the cellulose solvent.

## Supplementary Materials

The following are available online at www.mdpi.com/1996-1944/10/5/526/s1. Figure S1, ATR-FTIR characterization of modified cellulose under different temperature. Figure S2, ATR-FTIR characterization modified cellulose at different reaction time. Figure S3 Pictures of the cellulose and succinylated cellulose. Table S1, The degree of substitution (DS) of succinylated cellulose.
