# Supplementary material for: Modification of Cellulose with Succinic Anhydride in TBAA/DMSO Mixed Solvent under Catalyst-Free Conditions
Source: Materials (Basel). 2017 May 12;10(5):526. doi: 10.3390/ma10050526 (PMC5459046; doi:10.3390/ma10050526)
Supplement: Supplementary file 1 [file materials-10-00526-s001.pdf]

Supplementary Information

Modification of Cellulose with Succinic Anhydride in TBAA/DMSO Mixed Solvent Under Catalyst-Free Conditions

Ping-Ping Xin, Yao-Bing Huang, Chung-Yun Hse, Huai N. Cheng, Chaobo Huang and Hui Pan

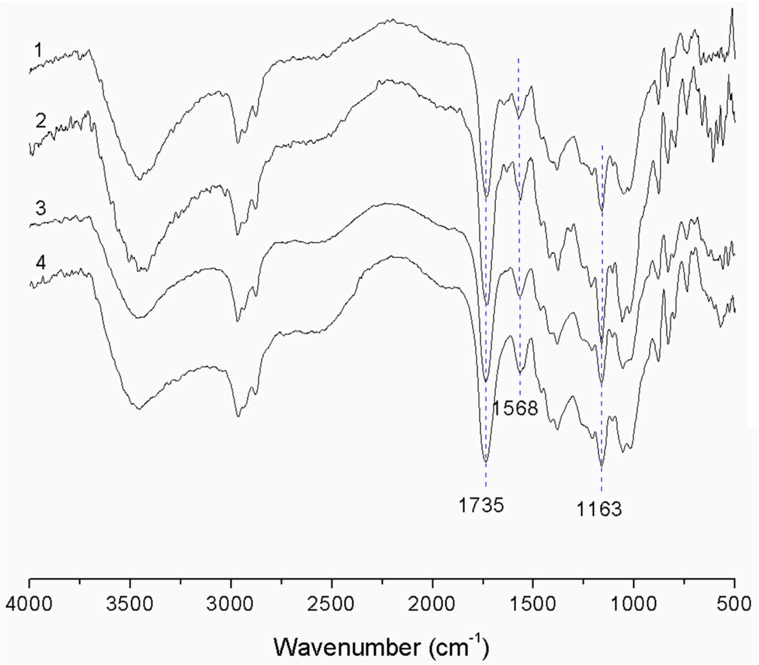

Figure S1. ATR-FTIR spectra of succinylated cellulose prepared at 20 °C (spectrum 1), 40 °C (spectrum 2), 60 °C (spectrum 3), 80 °C (spectrum 4).

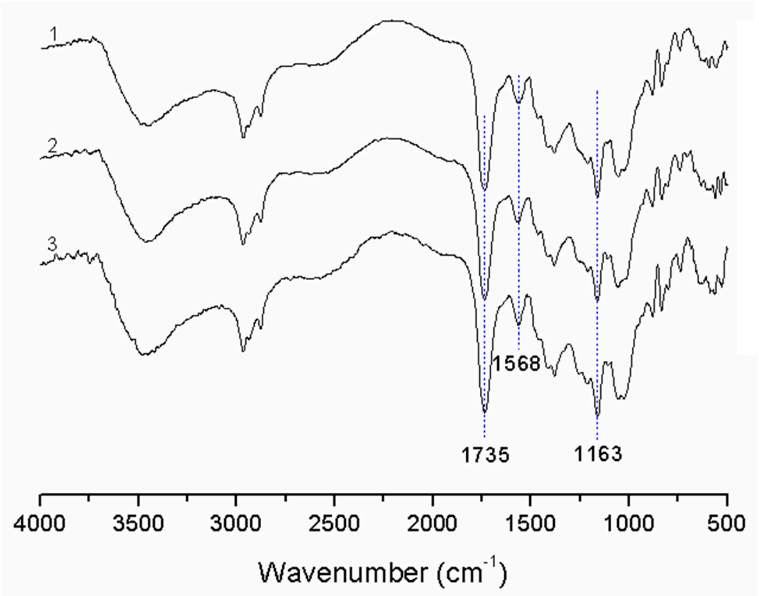

Figure S2. ATR-FTIR spectra of succinylated cellulose prepared at 60 °C for 30 min (spectrum 1), 60 min (spectrum 2) and 90min (spectrum 3).

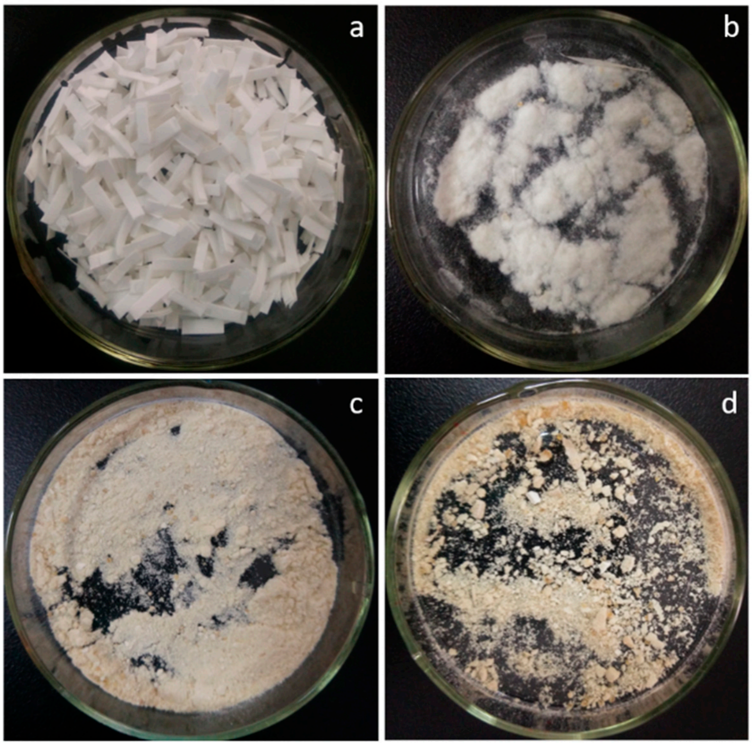

Figure S3. Pictures of unmodified cellulose and succinylated cellulose: (a) original filter paper and modified cellulose in TBAA/DMSO mixed solvents with the TBAA dosage of (b) 5.0 wt%, (c) 10.0 wt% (d) 12.5 wt%.

Table S1 The degree of substitution (DS) of succinylated cellulose.

	**Succinoylation conditions**[a]				
**Sample**	W_{TBAA} **(wt%)**	**Succinic anhydride / anhydroglucose in cellulose (mol:mol)**	**Temperature (°C)**	**Raction time (min)**	**DS**
1	5	2:1	60	60	0.337
2	5	4:1	60	60	0.595
3	5	6:1	60	60	0.698
4	7.5	2:1	60	60	0.487
5	7.5	4:1	60	60	0.719
6	7.5	6:1	60	60	0.880
7	10	2:1	60	60	0.525
8	10	4:1	60	60	1.002
9	10	6:1	60	60	1.191
10	12.5	2:1	60	60	0.491
11	12.5	4:1	60	60	0.815
12	12.5	6:1	60	60	1.036
13	10.0	4:1	20	60	0.933
14	10.0	4:1	40	60	0.960
15	10.0	4:1	80	60	1.000
16	10.0	4:1	60	30	1.049
17	10.0	4:1	60	90	1.064

[a] concentration of cellulose in ionic liquid/co-solvent mixed solvent during dissolution was 2.0 wt%
